# The effect of post-traumatic-stress-disorder on intra-operative analgesia in a veteran population during cataract procedures carried out using retrobulbar or topical anesthesia: a retrospective study

**DOI:** 10.1186/s12886-017-0479-2

**Published:** 2017-06-07

**Authors:** Yuna Rapoport, Laura L. Wayman, Amy S. Chomsky

**Affiliations:** 1 VA Tennessee Valley Healthcare System, Nashville, Tennessee USA; 2 Vanderbilt Eye Institute, Nashville, Tennessee USA; 3 Massachusetts Eye and Ear Infirmary, 243 Charles Street, Boston, MA 02114 USA

**Keywords:** Post-traumatic stress disorder, Anxiety, Cataract surgery, Anesthesia, Pain control, Vulnerable population

## Abstract

**Background:**

A growing proportion of veterans treated at the Veterans Health Administration (VA) have a history of post-traumatic-stress-disorder (PTSD), and there exists a higher rate of PTSD amongst veterans than the general population. The purpose of this study is to determine the correlation between PTSD and intra-operative analgesia, intra-operative time, and anesthesia type for cataract surgery in a veteran population. Secondary objectives are to determine if patient age, and first or second eye surgery affect intra-operative pain control or are correlated with type of anesthesia modality.

**Methods:**

A retrospective study of 330 cataract surgeries performed by resident physicians between January and September 2012 at the Veterans Affairs Medical Center Tennessee Valley Healthcare System, Nashville and Murfreesboro Campuses was completed. Three hundred and thirty veteran patients were selected if their cataract surgery was performed between January and September 2012. Combined cases were excluded. The primary outcome evaluated was intra-operative analgesia. Secondary outcomes included history of post-traumatic-stress-disorder, anesthesia type, first or second eye, pain control, intra-operative heart rate and blood pressure, age, and case complexity. Data was analyzed using an unpaired two-sample Welch’s t-test assuming unequal variance and Z test of comparison of proportions.

**Results:**

Patients with post-traumatic-stress-disorder reported higher pain scores, had longer operative times, and were more likely to have received a retrobulbar block. Operative time was not associated with an increased pain score, irrespective of anesthesia type, when controlled for PTSD. Complex cases had longer operative times, more sedation, and higher pain scores. *P* < 0.05 was used consistently.

**Conclusions:**

Post-traumatic stress disorder and anxiety are more prevalent in the veteran population. Our data suggests that a history of post-traumatic-stress-disorder was correlated with higher pain scores, longer operative times, and with having received a retrobulbar block. Patients without a history of PTSD were more likely to have received topical anesthesia with or without sedation. The veteran population requires more sedation to allay anxiety and perceptions of discomfort, which may account for longer surgical times. The veteran population is a special population and it is important to investigate how PTSD in the veteran population affects intra-operative analgesia.

## Background

The rates of post-traumatic-stress-disorder (PTSD) are higher amongst the veteran population than the general United States population. Amongst all American men, estimated lifetime prevalence of PTSD is 3.6% according to the National Comorbidity Survey Replication (NCS-R) [1], while the rates of PTSD in veterans is significantly higher and varies depending on the specific war during which the veteran fought. According to the National Vietnam Veterans Readjustment Study (NVVRS) [2], the estimated lifetime prevalence of PTSD was 30.9% for men. The prevalence of PTSD in a sample of Gulf War Veterans was found to be 12.1% [3]. The prevalence of PTSD among veterans deployed during Operation Enduring Freedom and Operation Iraqi Freedom (Afghanistan and Iraq) was estimated to be 13.8% [4]. In both the Gulf War trial by Kang et al. [3] and the Operation Enduring Freedom trial by the RAND corporation [4], PTSD was assessed using the PTSD checklist (PCL) [5].

Cataract surgery is one of the most common surgeries performed in the Veterans Health Administration (VA) [6]. In a given year, the VA performs more than 49,000 cataract surgeries, which represent over 11% of all surgeries performed in VA facilities [7]. Given the high prevalence of PTSD among the veteran community, it is important to consider the presence of any type of psychiatric history when planning for anesthesia and surgery. To the best of our knowledge, this is the first study to examine the effect of PTSD on intra-operative analgesia and cataract surgery thus far.

There are many types of anesthesia that are currently utilized during cataract surgery in the VA. These include general anesthesia, retrobulbar block (RBB), peribulbar block (PBB), sub-Tenon’s injection, and topical anesthesia with or without perioperative intravenous sedatives [8]. In studies performed thus far, there has been no definitive conclusion as to which type of anesthesia modality provides the most comfort intra-operatively in the general population. While some studies report patients experience more pain with retrobulbar block [9], most studies suggest that retrobulbar block provides better pain relief [10–13]. Yet others suggest sub-Tenon’s injection provides better anesthesia than topical [14] and than peribulbar [15]. Some studies suggest that when given the choice, patients prefer topical anesthetic to retrobulbar block [16], while another reported that patients prefer RBB to topical [13]. Among those receiving topical anesthesia, use of sedatives and opioids reduce pain during surgery [17]. Several randomized control trials found no difference between RBB and topical [18–20]. A meta-analysis found no significant differences between RBB vs. PBB (Table 1) [21].

**Table 1:** Summary of literature review of anesthesia modalities and pain during cataract surgery

Study reference	Study design	Cases (n)	Methods	Anesthesia modalities compared	Key findings	Other conclusions
Jacobi PC, et al. (2000) [14]	RCT	476	2 institutions, risk factors for complicated cases inclusion criteria: exfoliation syndrome, uveitis, posterior synechia, phacodonsesis, previous intraocular surgery	RBB (238) vs. topical (238)	- Intraoperative patient report of pain similar between the two - Higher patient preference for topical (*p* = 0.01)	- Vitreous loss lower in topical group (*p* < 0.05) - Intraoperative difficulty lower in RBB (measured by surgeons) (*p* < 0.01)
Gombos K, et al. (2007) [9]	RCT	115	June- Sept 2004. Exclusion criteria: other eye disease, previous surgery on same eye, increased risk of complication, poor pupil dilation	RBB (57) vs. topical (58)	- RBB provides better pain control (*p* < 0.01) - Systolic BP lower in RBB (*p* < 0.05) - Pain sensitivity higher in younger patients and patients with higher initial cortisol and noradrenaline serum levels	- RBB and topical both appropriate - Caution with topical with susceptible groups - Patients remembered pain more than actually indicated intraoperatively in both methods - SBP and DBP increased in the topical group and decreased in the RBB group (*p* < 0.05)
Patel B, et al. (1996) [18]	RCT	138	Assigned to groups by permuted block restricted randomization; no exclusion criteria	RBB (69) vs. topical (69)	- More discomfort during administration of topical anesthesia and postoperatively with topical (*p* < 0.05)	- No difference in pain - No difference in surgical conditions - Chemosis, subconjunctival hemorrhage, eyelid hemorrhage, retrobulbar hemorrhage only in RBB - Eyeball movement, squeezing of eyelids more common in topical; neither posed a problem to the surgeon
Patel B, et al. (1998) [10]	RCT	99	Prospectively assigned by permutated block restricted randomization; no exclusion criteria	RBB (45) vs. topical (45)	- Intraoperative operative conditions better in RBB (*p* < 0.05) - Better pain control with RBB (*p* < 0.05)	- No difference in postoperative discomfort - More pain in all cases after 20 min
Fazel M, et al. (2008) [16]	RCT	564	Consecutive adult patients presenting to Matini Hospital of Kashan University of Medical Sciences from Feb. 2007- March 2008 assigned via computer-generated number table; exclusion criteria: any other ocular pathology, anxiety history, difficulty laying flat, hearing impairment, cough	RBB (235) vs. topical (238)	No difference in pain between the two modalities	- No difference in blood pressures or heart rates - No differences between phaco time, age, sex, and postoperative visual acuity
Zhao LQ, et al. (2012) [11]	Meta-analysis	2205 patients from 15 RCTs	Cochrane Library, PubMed, EMBASE databases until 2010; no exclusion criteria	RBB/PBB (1121) vs. topical (1084)	- RBB/PBB provided better pain control (*p* < 0.05)	- Topical had more frequent inadvertent eye movements and more need for supplemental anesthesia (*p* < 0.05) - No difference between intraoperative difficulties - Patients significantly preferred topical (*p* < 0.00001) - RBB/PBB had more anesthesia related complications (chemosis, periorbital hematoma, subconjunctival hemorrhage *p* < 0.05) - No difference in surgery-related complications
Kallio H, et al. (2001) [17]	RCT	317 eyes of 291 patients	Adult patients consecutively scheduled for cataract extraction from Aug 1998- Aug 1999 by 1 surgeon at the Helsinski Eye Hospital; randomized by the envelope method; no exclusion criteria	RBB/PBB (114) vs. topical (96) vs. combined (topical and propofol) (107)	- No difference in intraoperative pain, frequency of complications, or outcome measures - Fewer intraoperative difficulties in RBB/PBB vs. topical and vs. combined (*p* < 0.05)	- IV propofol added to topical did not improve operative conditions - Additional sedation was required more frequently in topical than RBB/PBB (*p* < 0.05) - Patients having bilateral surgery preferred combined over RBB (*p* < 0.050
Boezaart A et al. (2000) [8]	Randomized cross-over observational study	98 ASA I and II patients for bilateral surgery 1 week apart	Private clinic – patients randomized to receive topical one eye and PRBB in the other eye or vice versa	RBB + TA vs. TA + RBB	- RBB provided better pain control (*p* < 0.05) - Patients preferred RBB over topical - Most patients (98%) were not aware of RBB being injected	- Duration of surgery was similar (*p* = 0.06) - Intraoperative difficulty higher for topical (*p* < 0.050 - Surgical and anesthetic complications unremarkable for both
Nwosu S, et al. (2011) [7]	RCT	90	Consecutive adult patients presenting to Guiness Eye Center in Onitsha, Nigeria between March –June 2008, randomized by simple random sampling	RBB (35) vs. subconjunctival (55)	- RBB worse pain control (*p* > 0.05) - Difference in movement of the eyeball was more in subconjunctival group (*p* > 0.05) - Post-operative ptosis slightly more in RBB	-Subconjunctival technique needed less anesthetic volume and no need for ocular massage - More chemosis and subconjunctival hemorrhage with subconjunctival - Higher pain in RBB may be due to longer time of onset of action
Alhassan M, et al. (2008) [19]	Meta-analysis	1438 participants from 6 trials	Cochrane Library 2010, MEDLINE 1960-2010, and EMBASE search 1980-2010	RBB vs. PBB	- No difference in pain between the two modalities	- No difference of akinesia or need for further injections of local anesthetic - Chemosis more common after PBB - Lid hematoma more common after RBB - Retrobulbar hemorrhage occurred once after RBB
Davison M, et al. (2007) [12]	Meta-analysis	617 patients, 742 eyes from 7 RCTs	Cochrane Library 2006, MEDLINE 1990-2006, and EMBASE search 1990-2006	Sub-Tenon’s vs. topical	- Sub-Tenon’s provides better pain relief (*p* < 0.05)	- Results were statically significant but not necessarily clinically significant - pain scores skewed to lower end - Sub-Tenon’s caused more chemosis and sub-conjunctival hemorrhage - Posterior capsule tear and vitreous loss occurred more often in topical than sub-Tenon (not significant)
Briggs MC, et al. (1997) [13]	Retrospective	129	Two 4 week period audits in which every patient undergoing cataract surgery with one of these two methods was included (chosen by surgeon) at Royal Alexandra Hospital in Paisely, UK	Sub-Tenon’s (74) vs. PBB (55)	- Less pain in administration of Sub-Tenon’s - Fewer patients experienced scores of >3/10 in sub-Tenon’s (*p* < 0.05)	- Sub-Tenon’s provides better pain relief peri-operatively (*p* > 0.05) than PBB

*RCT* randomized control trial, *RBB* retrobulbar block, *PBB* peribulbar block, *SBP* systolic blood pressure, *DBP* diastolic blood pressure

There have been no studies examining which type of anesthetic choice is best suited for the veteran population or for patients with PTSD. Given the higher rates of PTSD, the veteran population should be studied as a separate special population that has unique characteristics and needs. The goal of the study was to determine the correlation of post-traumatic-stress-disorder (PTSD) with intra-operative analgesia, intra-operative time, and selection of different anesthesia modalities for cataract surgery in a veteran population. Secondary objectives were to determine if patient age, and first or second cataract surgery affect intra-operative pain control or are correlated with type of anesthesia modality. The data was subsequently analyzed to determine whether intra-operative time was correlated with different anesthesia modalities, patient age, and previous cataract surgery, and this was controlled for a history of PTSD.

To the best of our knowledge, this is the first study to evaluate the effect of history of anxiety and PTSD on intra-operative analgesia and operative time.

## Methods

After local Institutional Review Board approval was obtained, we conducted a retrospective chart review of 330 cataract surgeries performed by second year resident physicians in the Veterans Affairs Medical Center Tennessee Valley Healthcare System, Nashville and Murfreesboro Campuses, between January and September 2012. Cases were collected in chronological order in that calendar year until 330 cases were obtained. There were no exclusion criteria. Data collected included history of anxiety disorder, post-traumatic stress disorder, or depression, operative time in minutes, anesthesia type (topical with no sedation, topical with sedation, RBB or general), level of pain control obtained from anesthesia’s intra-operative assessment on a scale from 0 to 10, intra-operative average heart rate (HR) and blood pressure (BP), age, and complicated cases (need for iris manipulation, need for tryptan blue, capsular tear, or vitreous loss). Topical anesthesia was defined as one to two drops of tetracaine pre-operatively and intracameral lidocaine intra-operatively. Retrobulbar block consisted of a 3.5 mL mixture of 1.4 mL of 2% lidocaine (40% of injection), 0.75% bupivicaine (40% of the injection), and 10% of the injection hyaluronidase. Sedation consisted of midazolam, fentanyl, and/or propofol. The resident performing the surgery, under the supervision of the attending ophthalmologist, performed the RBB. Sedation was administered by the anesthesiologist or certified nurse anesthetist. As this was a retrospective study, patients were not randomized to a specific anesthesia type. All data is presented as mean and standard deviation. An unpaired two-sample Welch’s t-t test assuming unequal variance between groups was used to test the difference between group means. Welch’s t-test was used since it is insensitive to equality of the variances regardless of whether the sample sizes are similar. Z test of comparison of proportions was used to compare the percentages (proportion) of population receiving a specific anesthesia modality. A significance level of 95% was utilized. The data was independently sampled.

Of the 330 cases reviewed, 54 of patients had PTSD, anxiety, or depression, and 276 did not have PTSD. Fifteen received general anesthesia, 146 received RBB, and 169 received topical anesthesia. Amongst patients receiving topical anesthetic drops, 116 received adjunct intravenous sedation, and 53 did not receive any sedation. All 330 cases were male patients, ages 53-82. Two hundred and one of the cases were the patient’s first eye cataract surgery; 129 cases were the second eye surgery. Seventy-eight patients reported some level of pain, and 252 did not report any pain. Operative times ranged from 18 to 120 min. One hundred thirty-five cases were <45 min, and 195 cases were >45 min, 216 cases were <60 min, and 114 cases were >60 min. Seventy-two cases were complicated, and 258 were uncomplicated.

In the first part of the study, post-traumatic-stress-disorder (PTSD) was correlated with intra-operative analgesia, intra-operative time, and selection of different anesthesia modalities for cataract surgery. In the second part of the study, the cases were divided by time (< 45 min and >45 min, and (< 60 min and >60 min). Pain control, age, amount of sedation received, history of anxiety/ PTSD, and 1st or 2nd eye surgery were analyzed between the time groups. Pain scores between anesthesia modalities were analyzed between the time groups. The data was then controlled for complex cases with the same markers analyzed. Welch’s t-test was used for each comparison and Z test of comparison was used for proportions, and a significance level of 95% was utilized.

## Results

**Fig. 1:**
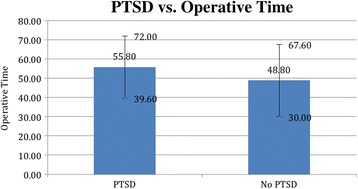
Patients with post-traumatic stress disorder/ anxiety cases have longer operative times. Bar graph of operative time (minutes) over case complexity. *Error bars* indicate standard deviation

**Fig. 2:**
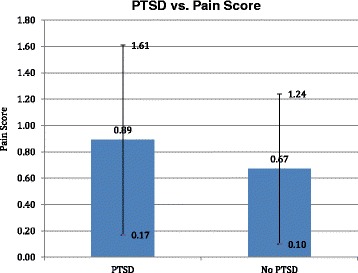
Patients with post-traumatic stress disorder/ anxiety have higher pain scores. Bar graph of pain scores over PTSD. *Error bars* indicate standard deviation

**Fig. 3:**
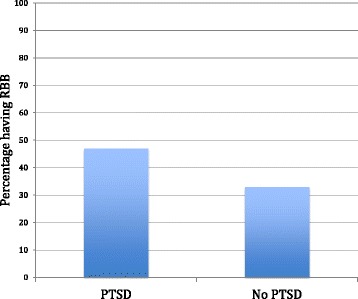
Patients with PTSD are more likely to have received a retrobulbar block. Bar graph of percentage of patients having RBB over PTSD

About sixteen percent (16.3%) of our veteran population had PTSD, correlating with prevalence of PTSD found in the veteran studies cited above) [2–4]. Patients with anxiety/ PTSD had significantly longer operative times (55.8 min +/− 16.2) than those without PTSD (48.8 +/− 18.8), mean difference 7, *p* < 0.01 (Fig. 1). Patients with PTSD were found to have higher pain scores (0.89 +/− 0.72) as compared with patients without PTSD (0.67 +/− 0.57), mean difference 0.22, *p* < 0.05 (Fig. 2). Those patients with PTSD, anxiety, or depression were more likely to have received RBB as their anesthesia modality (47%) as compared with patients without PTSD, (33%) *p* < 0.05 (Fig. 3).

**Fig. 4:**
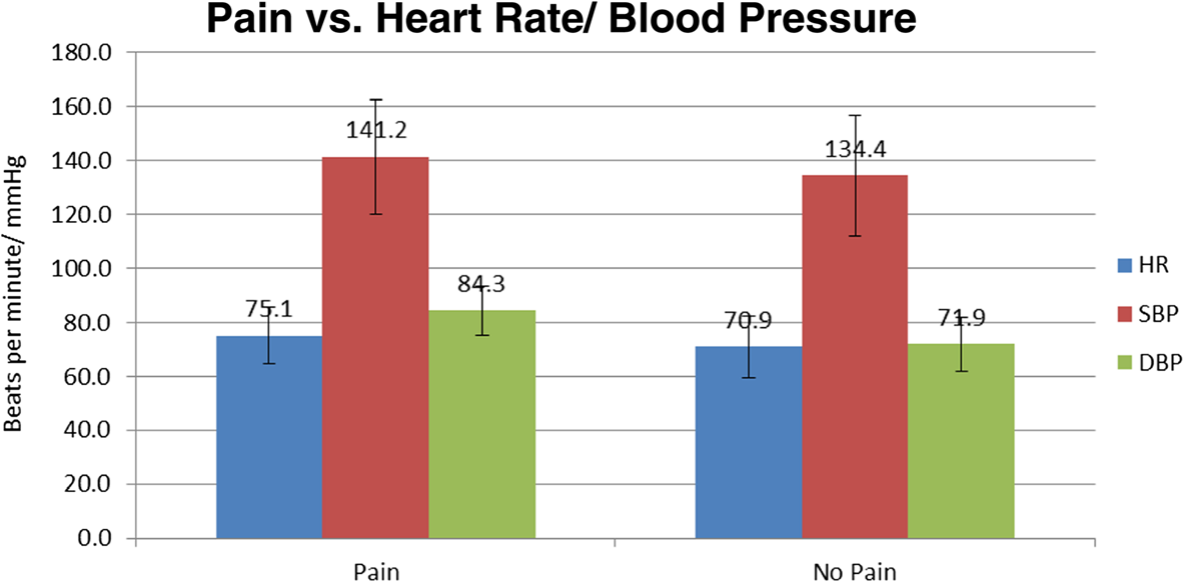
Heart rate, systolic blood pressure and diastolic blood pressure are higher in patients reporting pain. Heart rate, systolic blood pressure and diastolic blood pressure are higher in patients reporting pain as compared with patients reporting no pain. Bar graph of heart rate (beats per minute) and blood pressure (mmHg) over pain. *Error bars* indicate standard deviation

Heart rate (HR) was higher in patients who reported any pain (75.12 +/− 10.52), than those who reported no pain (70.90 +/− 11.28), mean difference 4.22, *p* < 0.01. Similarly, systolic blood pressure was higher in patients with pain (141.24 +/− 21.42) than those without (134.38 +/− 22.41), mean difference 6.86, *p* < 0.05; diastolic blood pressure was similarly found to be higher in patients with pain (84.28 +/− 9.20) than those without (71.89 +/− 10.19), mean difference 12.39, *p* < 0.0001 (Fig. 4). There was no difference in pain or type of anesthesia between 1st eye cataract versus 2nd eye cataract surgeries. Age was not associated with pain level or type of anesthesia.

**Fig. 5:**
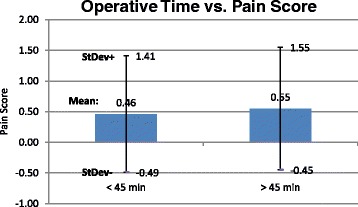
Operative time has no effect on pain score. Bar graph of pain score (mean) over operative time of cases under 45 min compared to over 45 min. *Error bars* indicate standard deviation

**Fig. 6:**
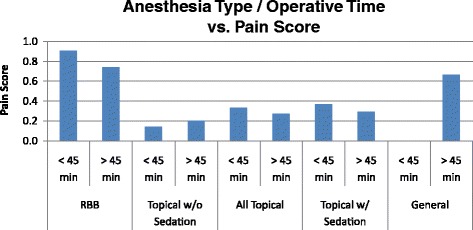
Operative time has no effect on pain score by anesthesia type. Bar graph of pain score (mean) over operative time of cases under 45 min compared to over 45 min, separated by anesthesia types

**Fig. 7:**
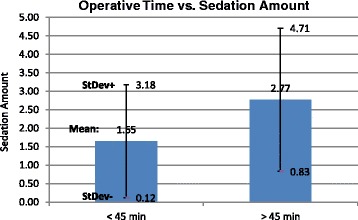
Longer cases receive significantly more sedation. Bar graph of sedation amount over operative time of cases under 45 min compared to over 45 min. *Error bars* indicate standard deviation

In Part Two, the data was analyzed while controlling for operative time and case complexity. Cases <45 min had a lower average pain score irrespective of anesthesia type while controlling for PTSD, but this did not reach statistical significance, *p* < 0.10 **(**Fig. 5). Within each anesthesia group, there was no statistically significant difference in pain scores between the time groups when divided into cases over and under 45 min (Fig. 6) and those over and under 60 min, when controlling for PTSD. Cases >45 min (2.77 +/− 1.94) received significantly more sedation than cases <45 min (1.65 +/− 1.53), mean difference 1.12, *p* < 0.0001 (Fig. 7). When controlling for time, patients with PTSD still reported higher pain scores than those without PTSD. This difference was present when cases were divided into those over and under 45 min and those over and under 60 min. All of the cases were performed by second year residents under the supervision of Attending surgeons and the residents were presumed to be of the same training level. Thus, the surgical times were not controlled by surgeon experience.

**Fig. 8:**
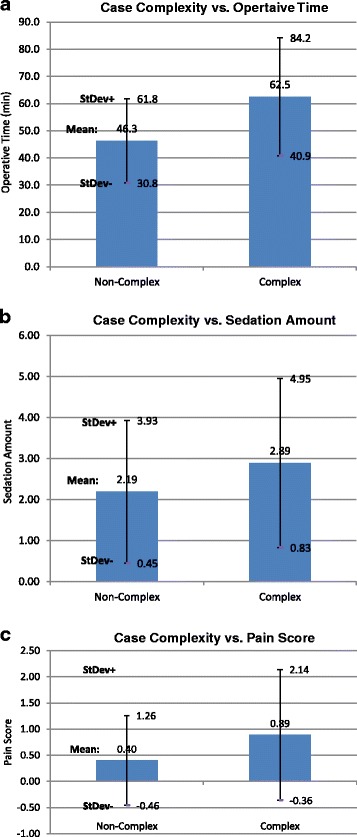
**a** Complex cases have longer operative times. Bar graph of operative time (minutes) over case complexity. *Error bars* indicate standard deviation. **b** Complex cases receive more sedation. Bar graph of sedation amount over case complexity. *Error bars* indicate standard deviation. **c** Complex cases have higher pain scores. Bar graph of pain score (mean) over case complexity. *Error bars* indicate standard deviation

Complex cases had longer operative times (62.52 +/− 21.66) than non-complex cases (46.30 +/− 15.51), mean difference 21.22, *p* < 0.0001 (Fig. 8a), and had more sedation (2.89 +/− 2.06) than non-complex cases (2.19 +/− 1.74), mean difference 0.70, *p* < 0.01 (Fig. 8b). Complex cases also had higher pain scores (0.89 +/− 1.25) as compared with non-complex cases (0.40 +/− 0.86), mean difference 0.49, *p* < 0.01 **(**Fig. 8c **)**.

There was no difference in age or 1st or 2nd eye surgery between those with and without PTSD, between anesthesia types and between the time groups.

## Discussion

Our study found that there was a significant correlation between a history of anxiety/PTSD and higher pain scores, longer operative times, and having had received a retrobulbar block. This population requires more sedation to allay anxiety and perceptions of discomfort, and this may account for longer surgical times. Patients with anxiety and PTSD having higher correlations with RBB may suggest that RBB may simply reflect anesthetic choice for specific patients. Patient comfort and expected operative difficulty were factors that influenced the selection of anesthesia at the time. In addition to anxiety/ PTSD, there are numerous other patient factors, both ocular and systemic, that could have contributed to anesthetic selection that were not directly measured in this study [22]. Such potential ocular factors include previous eye surgery, co-morbid ophthalmologic diseases, density of the cataract, the anterior chamber depth, the patient’s ability to fixate on the light, need for epinephrine and need for tryptan blue [23]. Patient systemic problems that could contribute to anesthetic choice not measured include the number and severity of baseline medical problems, presence of arthritis or back pain, number of medications and number of anxiolytics/ pain medications taken on a daily basis. Further studies analyzing anticipated difficulties could be investigated to see if any of these are confounding factors.

The correlation between intra-operative pain assessment and rise of intra-operative HR and BP could indicate pain, thus validating the pain assessment rubric. However, the rise in HR and BP is not specific and could represent increased sympathetic tone, which could be due to other reasons such as anxiety. Our data further showed that operative time was not associated with increased pain score, irrespective of anesthesia type, and irrespective of a history of PTSD. This may at least in part be due to longer operations receiving more sedation. This could suggest that the operative team prevents and responds well to patient pain.

In our study, there was no difference in pain scores between first or second eye surgeries, but in previous studies second eye surgeries were found to be more painful [24]. This is important to acknowledge when studying pain because frequency of second eye surgery has increased and now accounts for 35-40% of all cataract operations [25].

It is unclear whether results from this study can be extrapolated to a non-VA hospital because of demographic differences. The veteran population is unique in that it consists mostly of males of older age; studies have shown that both older patients and males have higher pain thresholds during cataract surgery [11]. In addition to having higher rates of PTSD, the VA population has a higher prevalence of chronic disease and worse self-reported health [26]. This may affect and influence the choice of anesthetic and operative times directly and indirectly in ways that were not measured in this study.

## Conclusions

Our study was the first to evaluate the effect of PTSD on intra-operative pain control, intra-operative time and anesthetic choice. The validity of this study is limited by its retrospective nature, sample size and other potential confounding variables not analyzed. Like all retrospective chart reviews, this study was limited by a lack of randomization and a lack of matched groups. There were no women included and thus there is a gender bias. However, by limiting the patient population, the recommendations are more pertinent to this specific group.

Several recommendations can be derived from this study. First, PTSD and anxiety are more prevalent in the veteran population. Next, PTSD is associated with longer operative times and is also associated with higher levels of intra-operative discomfort, which can include both pain and anxiety. Finally, RBB may be the preferred anesthetic technique for cataract surgery in this population because it completely obtunds sensory input. By investigating associations between post-traumatic-stress-disorder and pain, we can better anticipate the needs of our veteran patients. Anesthesia providers need to be cognizant of increased intra-operative sedation for patients with PTSD who have longer operative times and may subsequently require more sedation.

## Abbreviations

BP: Blood pressure
HR: Heart rate
PBB: Peribulbar block
PTSD: Posttraumatic stress disorder
RBB: Retrobulbar block
VA: Veterans Health Administration
